# Electronically switchable band tuned filtering power divider for Sub-6 GHz applications

**DOI:** 10.1038/s41598-026-50530-1

**Published:** 2026-05-14

**Authors:** Rohit Mathur, Dilip Kumar Choudhary

**Affiliations:** School of Electronics Engineering, Vellore Institute of Technology, Vellore, 632014 India

**Keywords:** Filtering power divider, Wideband, Narrowband, Isolation, 6G, Engineering, Physics

## Abstract

This work presents a compact electronically switchable filtering power divider (FPD) for Sub-6 GHz wireless front ends requiring adaptive and spectrum-flexible RF hardware. Conventional multi-band receivers rely on separate narrowband and wideband chains, resulting in increased loss, complexity, and circuit footprint. To overcome these limitations, a miniaturized band-tuned FPD is developed by integrating a folded-resonator bandpass filter with a Wilkinson-based power-divider core through a coupled-line capacitive interface. The resonator employs inverted S-shaped and modified C-shaped elements to achieve size reduction, while four PIN diodes embedded in a step-impedance resonating structure enable electronic switching between wideband and narrowband modes. In the diode-OFF state, the proposed structure realizes wideband operation centered at 2.9 GHz with a 34.4% fractional bandwidth, insertion loss near 1.4 dB, return loss better than 15 dB, and isolation better than 10 dB. While for the diode-ON state, the circuit offers narrowband response centered at 2.35 GHz, realizing return loss better than 20 dB, isolation better than 10 dB, within an electrical size of only 0.14λ₀ × 0.18λ₀. The results confirm that the proposed FPD provides equal power division with high isolation, and electronically selectivity.

## Introduction

The progress in modern wireless communication systems, particularly within the Sub-6 GHz spectrum, required high-performance front-end architecture that can dynamically adapt to varying channel bandwidths, frequency allocations, and signal conditions. Evolving communication standards such as 5G, LTE-Advanced, and beyond require compact, reconfigurable, and wideband hardware capable of operating over multiple frequency bands though maintaining high isolation, low insertion loss, and stable impedance matching^1,2^. To meet these strict requirements, front-end components such as power dividers and filters must evolve from static, single-function devices to multifunctional and electronically tunable systems.

**Fig. 1:**
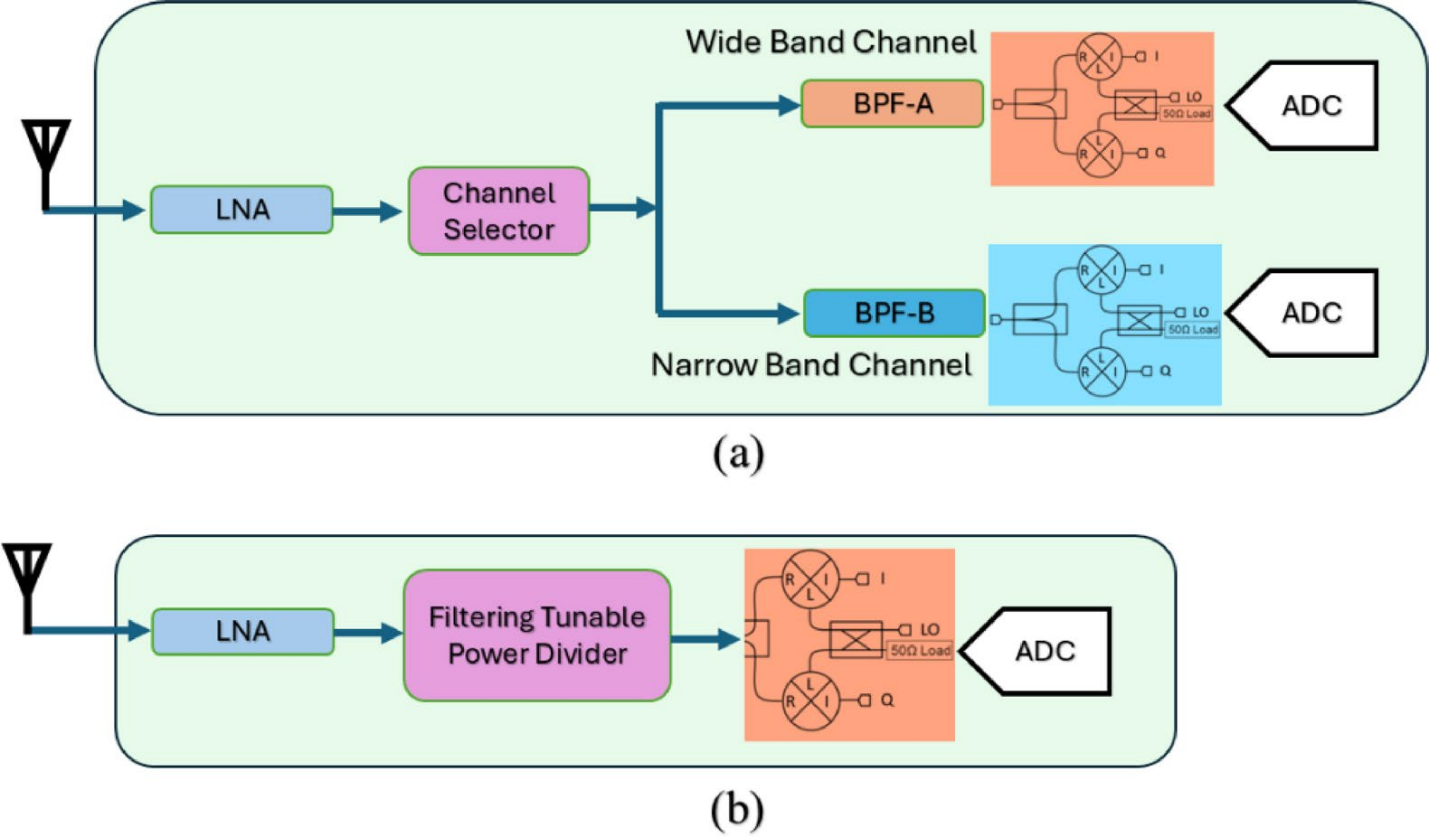
System block diagram of multi-band receivers (**a**) different channel to operate in narrow band and wide band (**b**) single channel using switchable Band Tuned Filtering Power Divider.

In conventional receiver designs as shown in Fig. 1a, different receiver chains are often employed for narrowband and wideband operation, where an RF switch network selectively connects the applicable path based on system requirements. This approach increases hardware complexity along with high insertion loss across different stage, and high system footprint. A more effective solution is the integration of band-selective filtering functionality directly within the power division network which enables a single compact circuit to simultaneously provide signal distribution and adaptive frequency selectivity.

Showing this approach in Fig. 1b, the Filtering Power Divider (FPD) has emerged as a promising approach capable of both power division and frequency-selective filtering. One way to do this is by integrating bandpass or lowpass filter sections within conventional power divider structures, such as the Wilkinson Power Divider (WPD), the FPD achieves simultaneous power-splitting and spectral-shaping characteristics. For instance, a bandpass filter was embedded in place of the quarter-wave transmission line (QTL) in a WPD to achieve a compact filtering response^3–5^, Earlier studies have demonstrated numerous single-band FPDs designed with WPD configurations, substrate-integrated waveguide (SIW) techniques, and dual-composite right/left-handed (D-CRLH) metamaterial structures^6–8^. while SIW-based designs have incorporated metamaterial-inspired structures to enhance frequency selectivity^7^. Moreover, D-CRLH-based bandpass elements have been utilized to realize miniaturized and high-performance FPDs^8,9^. Although dual-band FPDs have been reported in literature^10–13^, research on multiband or electronically tunable FPDs remains relatively limited^14–19^. The growing demand for adaptive and spectrum-efficient communication systems underscores the need for electronically switchable band-tuned FPDs capable of dynamically selecting operating bands without requiring multiple discrete receiver paths.

In recent decade Filtering power dividers (FPDs) have become integral to modern RF front ends due to their ability to combine power division with frequency-selective characteristics. In^20^ a generalized coupling-matrix–based framework that enables the systematic design of frequency-fixed, tunable, and multiband Wilkinson-type FPDs with canonical filtering responses has been reported. This work established a direct relationship between filter coupling matrices and divider topologies, providing a unified method for synthesizing both equal and unequal power division structures. Advances have also been made in reflectionless architectures^16^. Presents a tri-band filtering power divider by embedding a fork-type multi-mode resonator within a Wilkinson topology. The design achive multiple resonant modes from a single fork-shaped structure to get three independent passbands without cascading separate filters. Resonant frequencies are controlled by adjusting the resonator arm lengths and coupling gaps, while transmission zeros are introduced to enhance selectivity and isolation. In^21^, an all-port-reflectionless FPD that simultaneously achieves zero reflection at all ports while maintaining a predefined filtering response explained. Their formulation employs closed-form equations and offers wide port-to-port isolation without reliance on optimization. In related multifunctional developments,^22,23^ demonstrated a quasi-reflectionless tunable filtering antenna using a bandstop filter coupled with a patch antenna. Although antenna-focused, the work provides relevant insights into tunable resonator behavior and reflectionless signal control. Wideband unequal power division has been further expanded by^24^, which employed a triple-mode resonator to achieve large power-dividing ratios, broad matching bandwidth, and embedded filtering characteristics. The FPD described in^25^ makes use of an arbitrary power division ratio and a variable center frequency based on time-modulated resonators. it operate as an impedance transformer, filtering power divider, and isolator in a single circuit. In this work, a customizable power-dividing ratio with insertion loss control is presented. The Wilkinson power divider is first equipped with a narrow-band BPF based on the analogous circuit architecture. Numerous power-dividing ratios are produced by its insertion loss control components in^26^. In^27^, waveguide based FPD is designed using casecaded trisection based on crosscoupling to control the power division ratio. A controlled unbalanced power divider addressed a spoof plasmonic waveguide loading with varactors is suggested^28^. It is primarily made up of two branch single-sided plasmonic strips connected to a double-sided corrugated metal strip. Varactors are loaded in the coupling gap to modify the coupling coefficient, allowing flexible distribution of electromagnetic (EM) energy. Despite these advancements, electronically switchable band-tuned FPDs remain insufficiently explored, particularly for sub-6-GHz applications requiring reconfigurability with stable filtering and isolation performance.

## Configuration and design

This section describes the structure configuration and design evolution. The first phase is to design a Band Pass filter followed by power divider design with bandpass filter, and final design incorporates tunable filtering power divider.

### Design of band pass filter

**Fig. 2:**
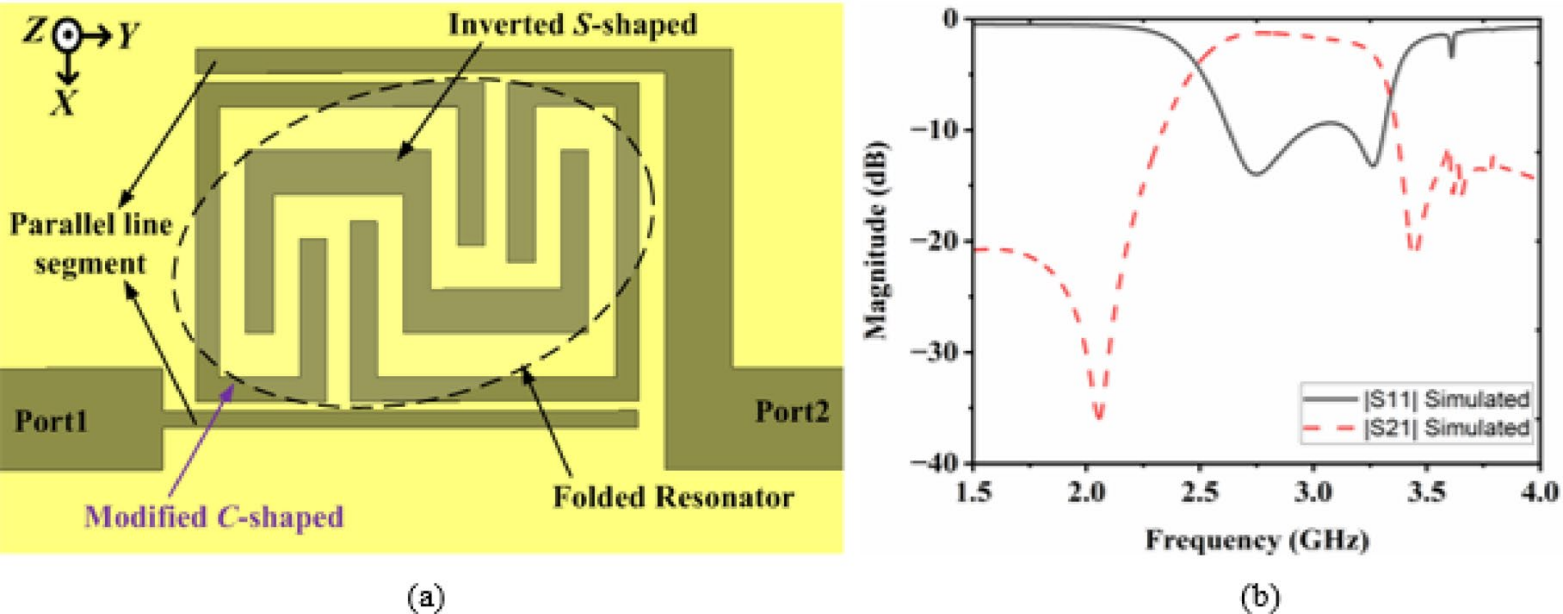
Bandpass Filter: (**a**) Structural layout (**b**) Scattering parameters.

The proposed bandpass structural layout is shown in Fig. 2a, employs folded resonator configuration and parallel line segments. The folded resonator consists inverted S-shaped and modified C-shaped structures to minimize the overall size without compromising the desired resonance characteristics. Compared to a conventional straight λ/4 resonator, the folded configuration reduces the physical footprint using meandering, while supporting the effective electrical path length required for resonance at the target frequencies. In addition to size reduction, the geometric folding introduces controlled electric and magnetic coupling between adjacent segments, supporting the creation of multiple transmission zeros through source–load and cross-coupling effects. These transmission zeros significantly enhance skirt selectivity and out-of-band suppression without requiring additional filter stages. The parallel-line segments introduce distributed capacitive and inductive effects that control the center frequency and impedance matching. Input and output feed lines connected at the two ends for signal excitation and extraction. When the input signal is applied, the energy is coupled between the folded resonators through both capacitive and inductive coupling. The coupling strength and resonant frequency can be controlled by adjusting: spacing between adjacent lines, line width, and total length of the meandered resonator.

The simulated scattering parameters of designed bandpass filter is shown in Fig. 2b. It is observed that passband having input reflection coefficient (S11) better than 12 dB with two poles and transmission coefficient 0.5 dB at centre frequency. The passband operating between 2.45 and 3.35 GHz having 3 dB fractional bandwidth of 31.03% at centre frequency 2.9 GHz. Three transmission zeros are located at 2.05 GHz, 3.45 GHz, and 3.65 GHz in this filter.

### Implementation of band pass filter with power divider

**Fig. 3:**
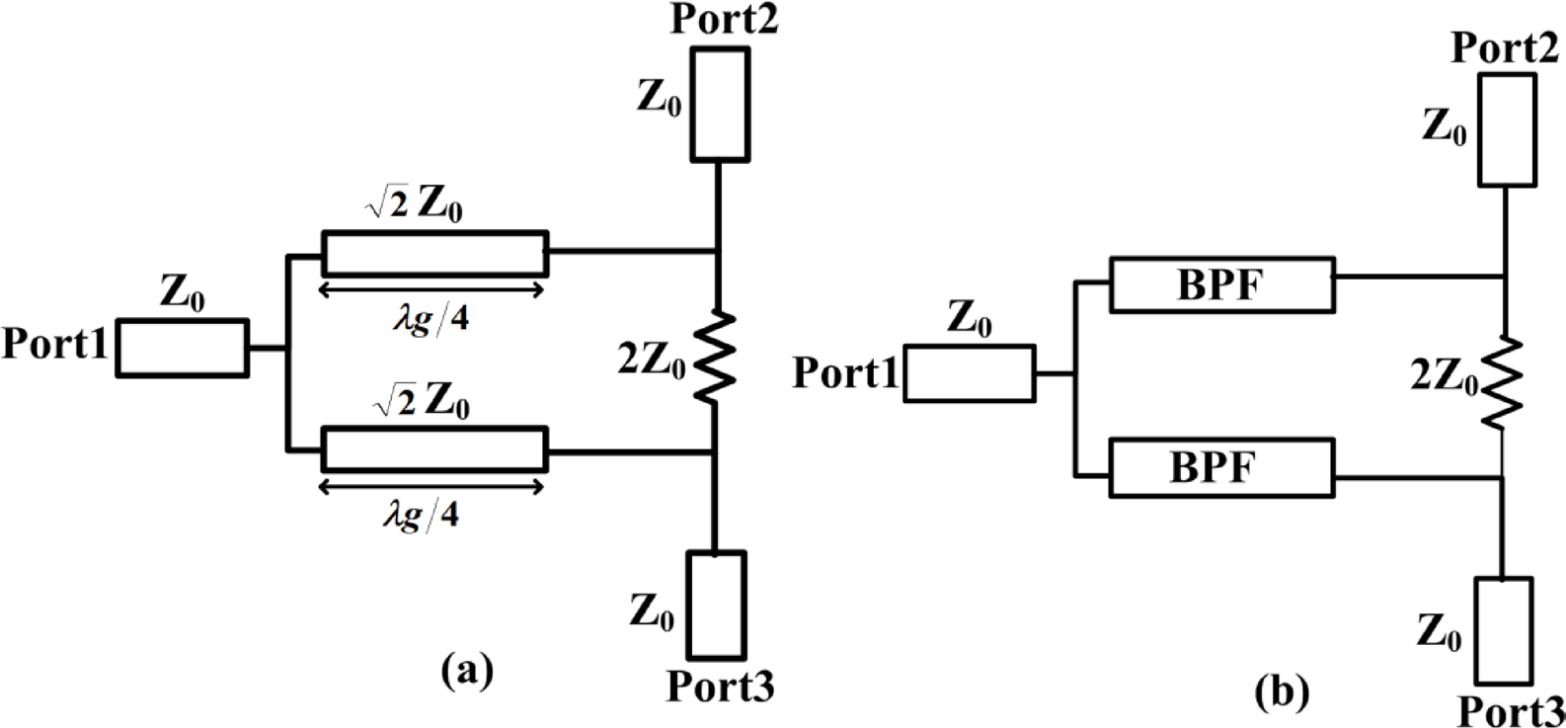
(**a**) Wilkinson power divider (**b**) proposed filtering power divider.

At First, a Wilkinson type power divider has been designed using two quarter-wave transmission lines (QW-TL) and an isolation resistor with values of Z_0_ and $\sqrt 2$Z_0_ respectively, where Z_0_ is the characteristics impedance of feedline. The Circuit schematic of Wilkinson power divider and designed filtering power divider (FPD) is shown in Fig. 3.

In FPD, QW-TL of power divider has interchanged with designed BPF structure by common capacitive coupled line segment. The Isolation resistor value of the Wilkinson power divider^4^ can be written as

1 $$\:R={Z}_{o}\left(\frac{({k}^{2}+1)}{k}\right)$$

**Fig. 4:**
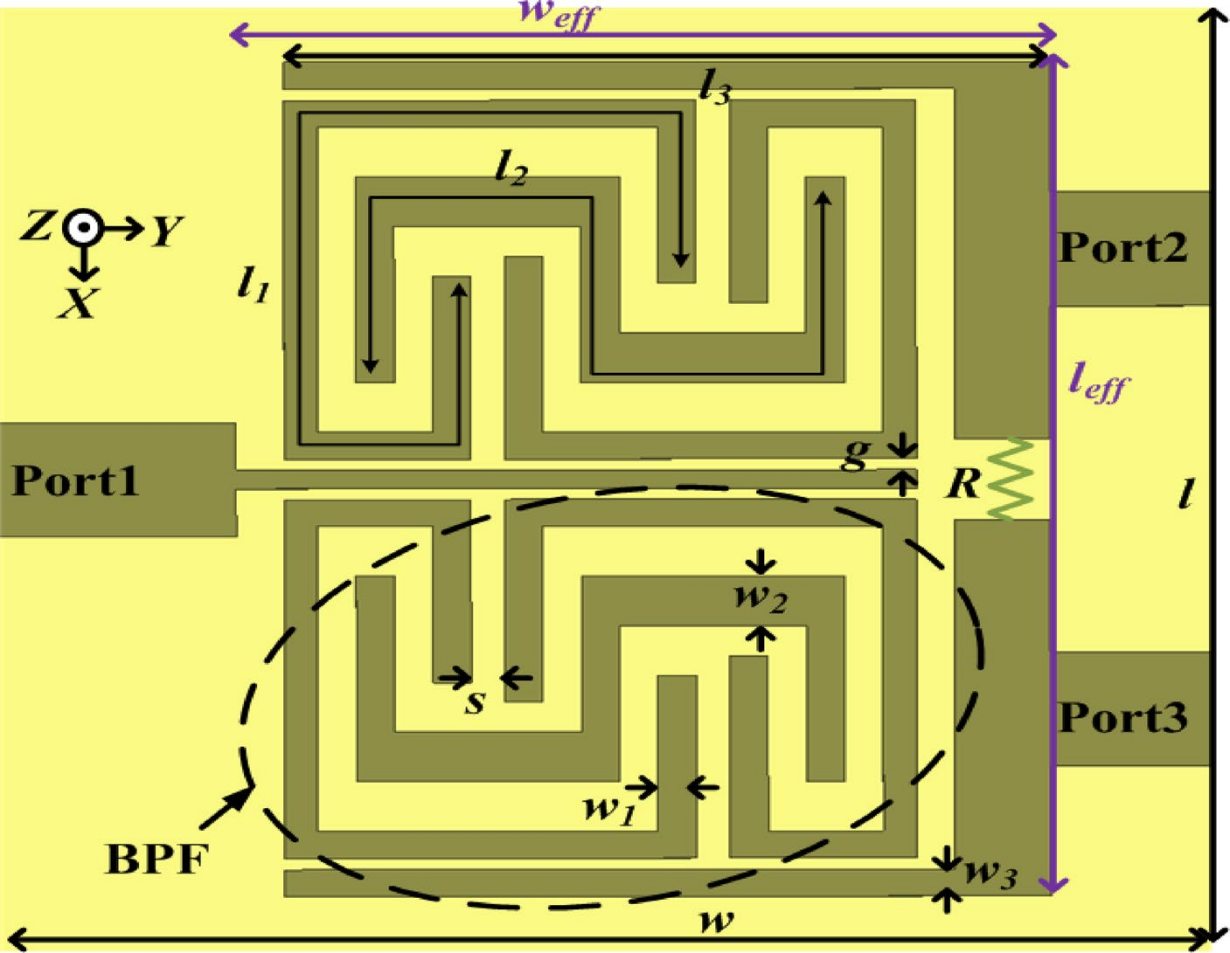
Layout of bandpass filtering power divider. [All dimensions are in mm: *l =* 25.0, *l*_*eff*_ *=* 21.8, *w =* 25.5, *w*_*eff*_ *=* 14.4, *l*_*1*_ *=* 31.2, *l*_*2*_ *=* 27.0, *l*_*3*_ *=* 16.0, *w*_*1*_ *=* 0.7, *w*_*2*_ *=* 1.3, *w*_*3*_ *=* 0.7, *g =* 0.3, and *s =* 0.7].

Here, *Z*_*o*_ represents source/load impedance, *k*^*2*^ represents the power ratio (for this work *k*=1as for equal power division), and *R* is the isolation resistance between output ports (port-2 and port-3) respectively. Therefore, for the proposed structure, *R*= 2*Z*_*o*_. The structural layout of proposed FPD consisting of a bandpass filter integrated with a power divider, is shown in Fig. 4. The filtering circuit consists folded resonators of inverted *S*-shaped and modified *C*-shaped elements with parallel line segment. Resonators are arranged in such way to provide strong capacitive coupling with compact structure. The physical length of modified C-shaped element is 31.2 mm, inverted S-shaped length 27.0 mm, and parallel line segment is 16.0 mm long.

### Design of tunabel filtering power divider

**Fig. 5:**
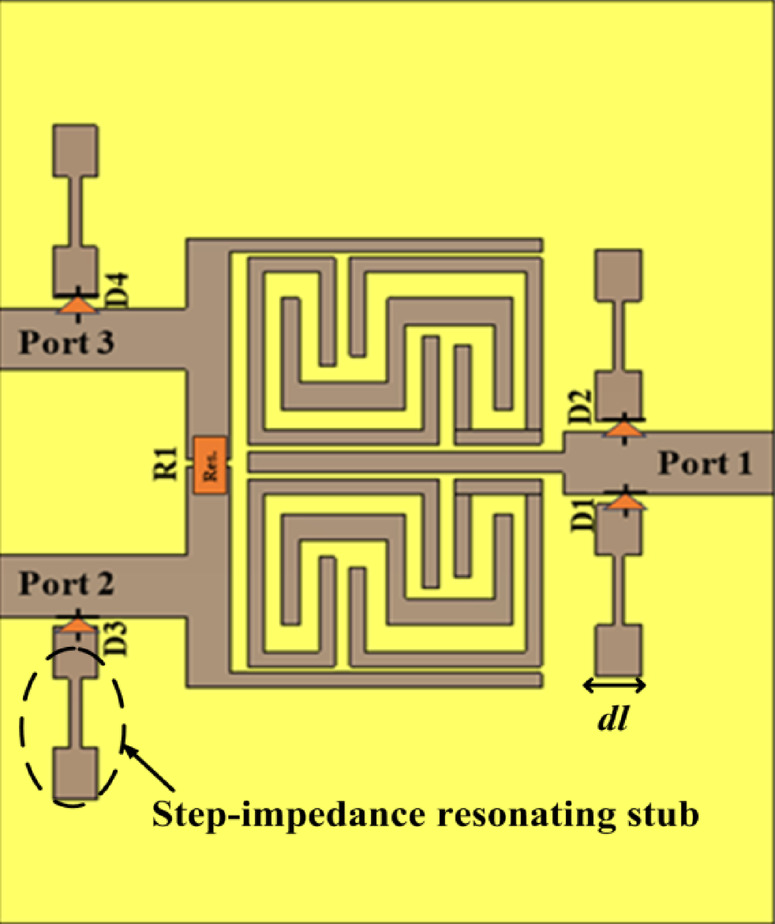
Layout of the designed tunable filtering power divider.

 The initial design of the Wilkinson power divider, as discussed in Sect. 2.2, incorporates inverted S-shaped and modified C-shaped resonators arranged to provide strong capacitive coupling within a compact structure.The geometric configuration of the final filtering circuit is shown in Fig. 5^29^. It includes a filtering step-impedance resonating stub, an isolation resistor R1, and four PIN diodes (D1, D2, D3, and D4). These PIN diodes are fundamental for band-tuning operations.

**Fig. 6:**
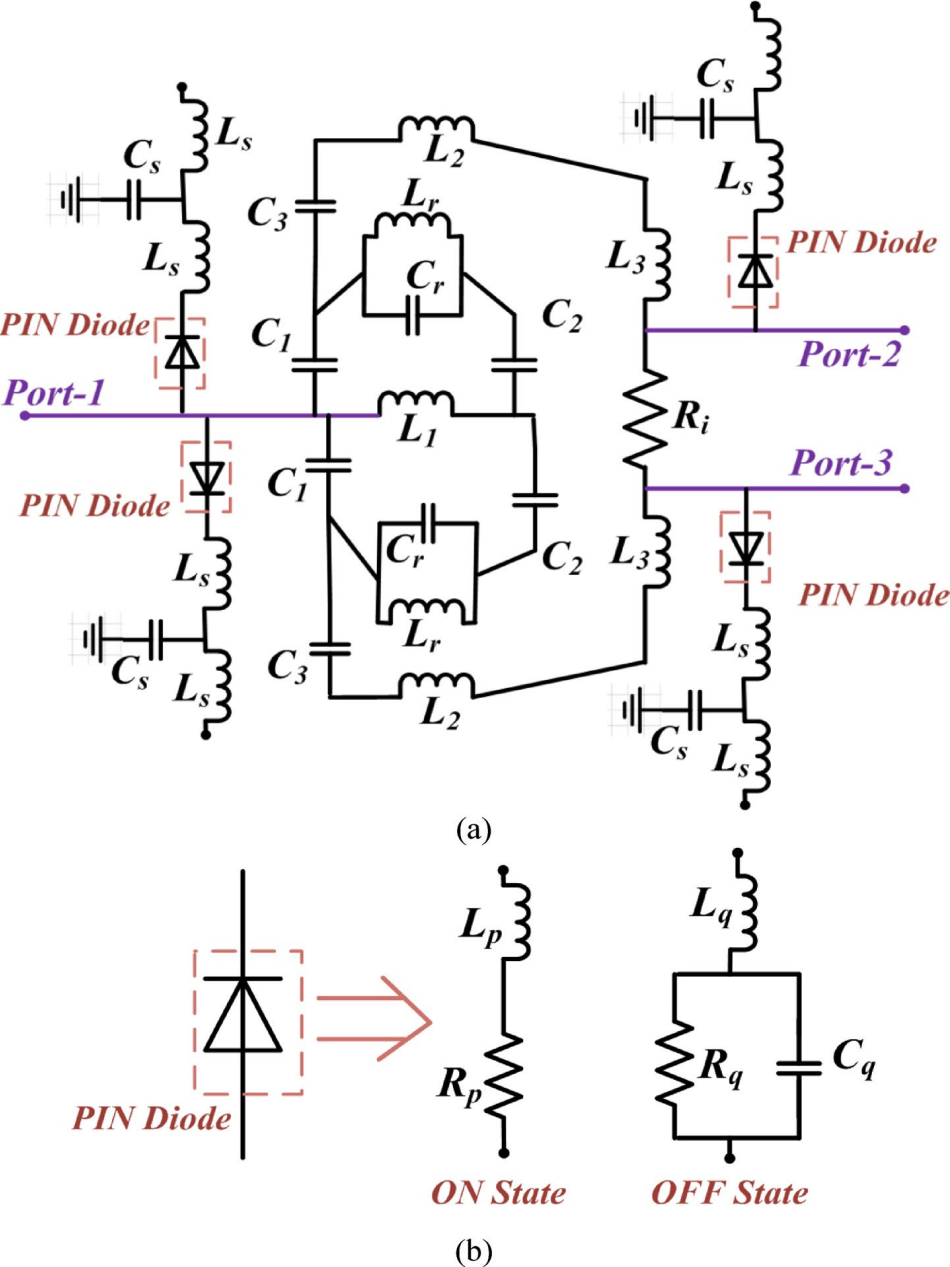
Equivalent circuit model of proposed band tunable filtering power divider.

The lumped equivalent circuit model of proposed tunable wideband/narrowband filtering power divider is depicted in Fig. 6a and b. It enables the selective bandwidth and controlled isolation among three ports as Port-1, Port-2, and Port-3 using PIN diodes and a combination of lumped inductive and capacitive elements. On each port a duo of series inductors (L_s_) and shunt capacitors (C_s_) signify the parasitic and matching characteristics of the stepped impedance resonator. The transition from narrowband to wideband operation is facilitated by PIN diodes integrated near each port. Certain RF routes can be opened or closed by biasing these diodes ON or OFF, allowing for dynamic control of the signal flow between the ports. The folded bandpass resonator consists inductors L₁, L₂, and L₃ provides magnetic-coupling routes between the ports. The coupling capacitors C₁, C₂, and C₃ enhance inter-port isolation, adjust the operating frequency, and modify the impedance response. The parallel resonant tanks formed by the L_r_C_r_ branches offer high impedance at resonance, preventing unwanted coupling routes and improving port isolation. The following are circuit schematic values using the ADS circuit simulator: *L*_*1*_ = 15.5, *L*_*2*_ = 2.7, *L*_*3*_ = 21.6, *L*_*s*_ = 8.17, *L*_*r*_ = 3.3, *L*_*p*_ = 38.1, *L*_*q*_ = 44.0, *C*_*1*_ = 0.32, *C*_*2*_ = 10.3, *C*_*3*_ = 4.3, *C*_*s*_ = 0.9, *C*_*r*_ = 3.1, *C*_*q*_ = 9.6, *R*_*1*_ = 100, *R*_*p*_ = 3.4, and *R*_*q*_ = 3.4. Here all inductors, capacitors and resistors values are in *nH*, *pF*, and Ohm, respectively.

**Fig. 7:**
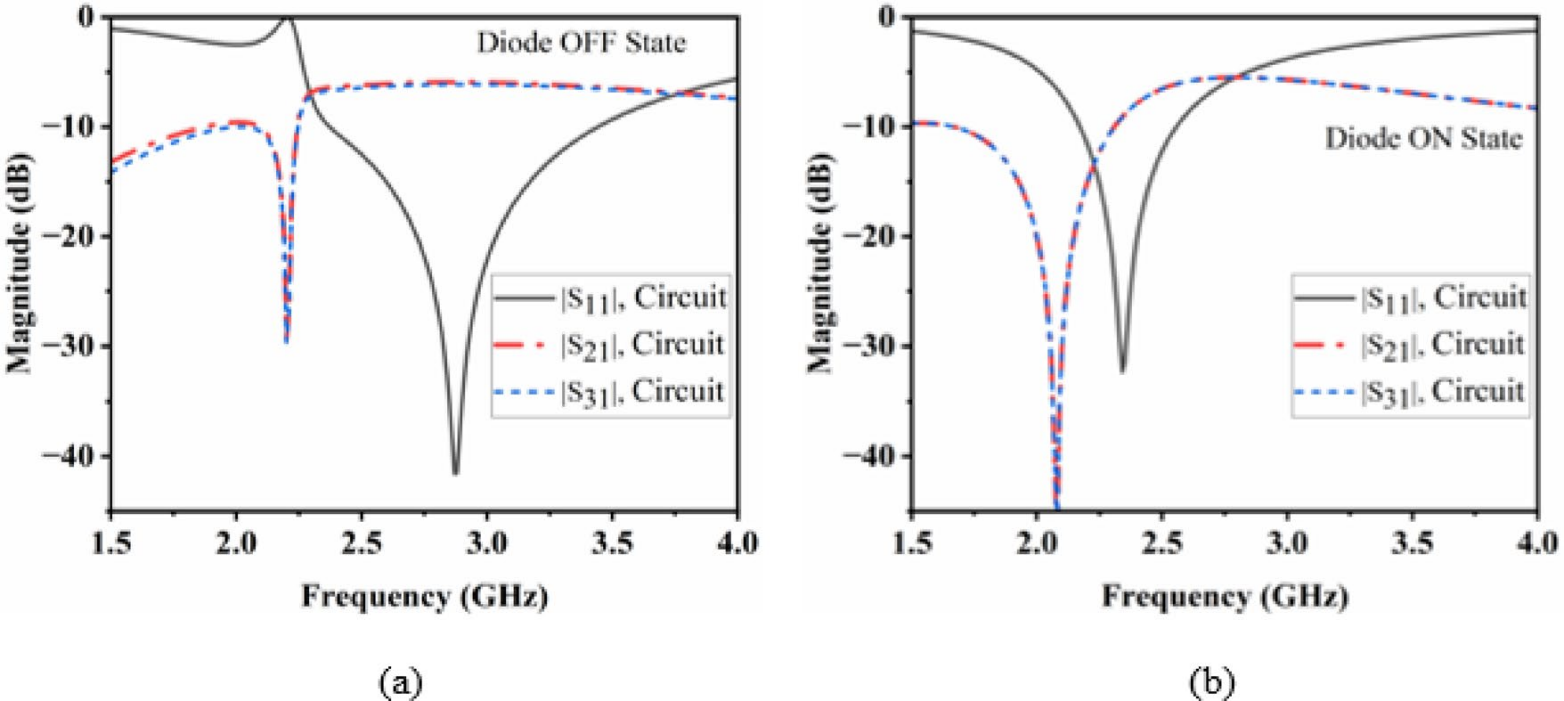
Circuit simulated S-parameters tunable FPD: (**a**) Diode-OFF and (**b**) Diode-ON conditions.

**Table 1:** Summary of diode State versus operating band.

Diode state	Band of operation
All diodes (D1, D2, D3, and D4) are ‘OFF’	Wide band state (2.4 to 3.4 GHz)
All diodes (D1, D2, D3, and D4) are ‘ON’	Narrow band state (2.3 to 2.4 GHz)
Any other configuration	Narrow band state of not interest

The proposed tunable FPD circuit simulator s-parameters under two diode-biasing scenarios are shown in Fig. 7. The PIN diode is reverse-biased in the Diode-OFF state, creating a high-impedance condition that successfully inhibits the signal flow through the chosen branch provides wider banwidth. When the path is disabled, the associated S-parameter response shows great isolation between the ports, with S₂₁/S₁₂ remaining considerably attenuated across the operational band and the return loss being consistent, suggesting that the input matching is not significantly affected. On the other hand, the diode is forward-biased and offers a low-impedance channel when it is in the Diode-ON state provides narrow bandwidth, which permits transmission along the specified port pair. The desired switching action is confirmed by the S-parameters in this condition, which exhibit good transmission (low insertion loss) and a discernible decrease in isolation.

**Fig. 8:**
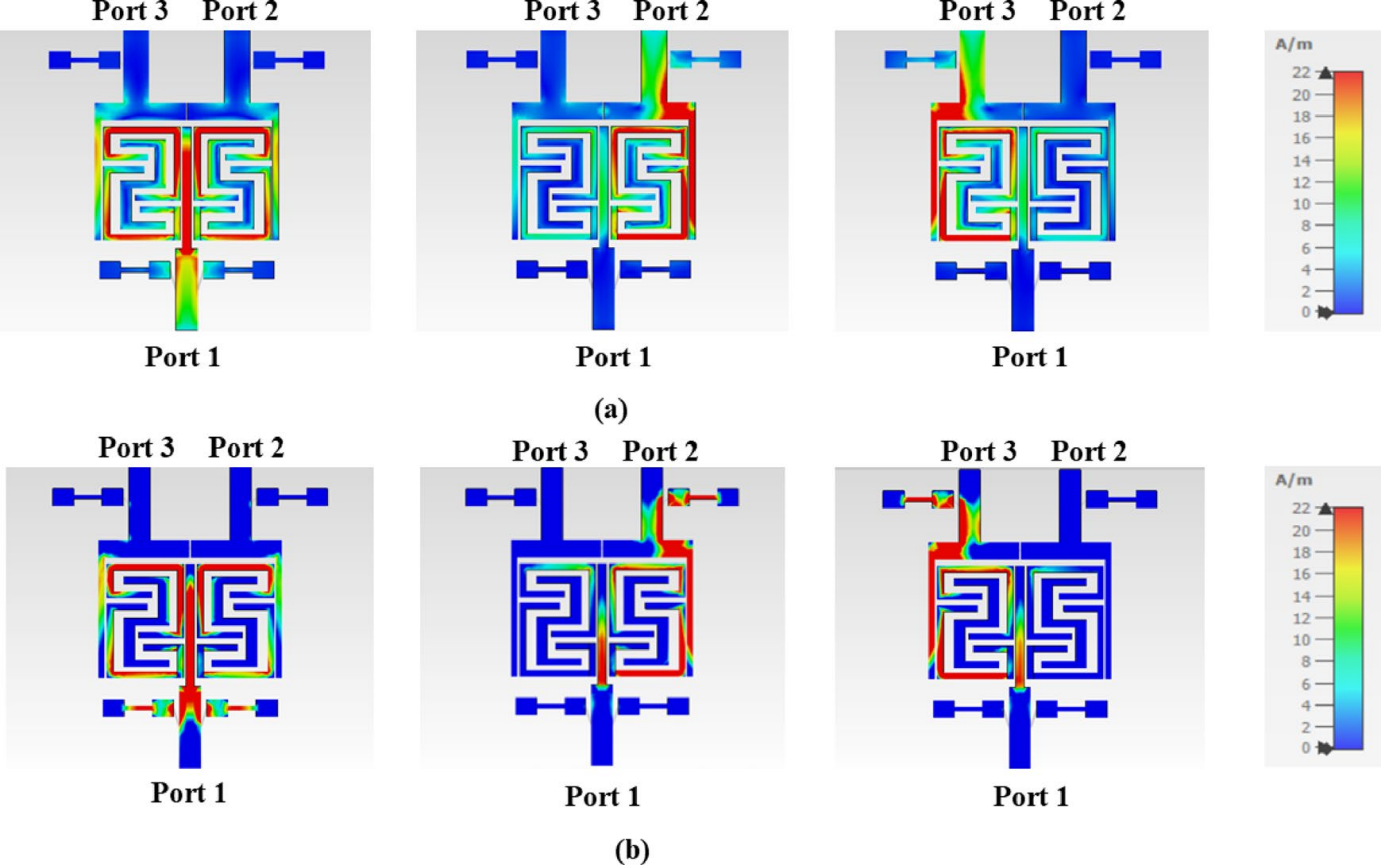
Surface Current distribution over each port for Diode ON State (**a**) for 2.35 GHz (**b**) for 2.9 GHz.

In the diode-OFF state, the PIN diodes behave as open circuits, leaving the stepped-impedance resonating stubs electrically disconnected. Consequently, the excitation at Port 1 predominantly couples into the folded S- and C-shaped resonators, establishing a wideband current distribution across both resonant arms.The uniform current flows toward Ports 2 and 3 as conventional Wilkinson-type power division, with minimal suppression of out-of-band energy. However, in Diode ON state narrowband filtering has been performed. Figure 8 illustrates the simulated surface-current distributions of the proposed filtering power divider under diode-ON conditions at two different frequencies, highlighting the transition from weak to strong resonance of the activated step-impedance stubs. Further any other confugration of diode make FPD in narrow band state with poor isolation and transmission coefficient also summerized in Table 1.

The surface-current distribution in the diode-ON state at 2.35 GHz and 2.9 GHz can be interpreted using a coupling-mode perspective, where the resonant behaviour of the structure is governed by how individual resonators exchange energy and how strongly each mode couples to the feed and output ports. At 2.35 GHz, the step-impedance stubs activated by the ON-state diodes remain far from their natural resonance; consequently, their coupling strength to the main line is weak, and they do not significantly participate in energy exchange. The electromagnetic energy injected at Port 1 therefore excites a mixture of weakly coupled higher-order modes in the folded S- and C-shaped resonators, leading to a diffuse current distribution with no dominant mode. This indicates that the resonant structure effectively rejects energy at this frequency in the ON state, consistent with the divider’s selective transmission and narrow-band behaviour. At 2.9 GHz, however, the stubs become strongly resonant, and their coupling to the feedline sharply increases, causing them to dominate the energy flow. The localized, high-intensity currents observed on the stub network reflect the activation of a single strong resonant mode, while the folded resonators remain largely inactive. Because the resonant energy is tightly confined to the stub path, only specific coupling routes toward the output ports remain active, enabling selective reinforcement of the desired narrowband and producing the enhanced isolation measured at this frequency. Thus, the contrasting current distributions at 2.35 GHz and 2.9 GHz arise from a shift in the dominant resonant mode and its coupling pathways, illustrating how diode-controlled resonance governs the divider’s broadband and narrowband filtering behaviour.

## Result analysis

**Fig. 9:**
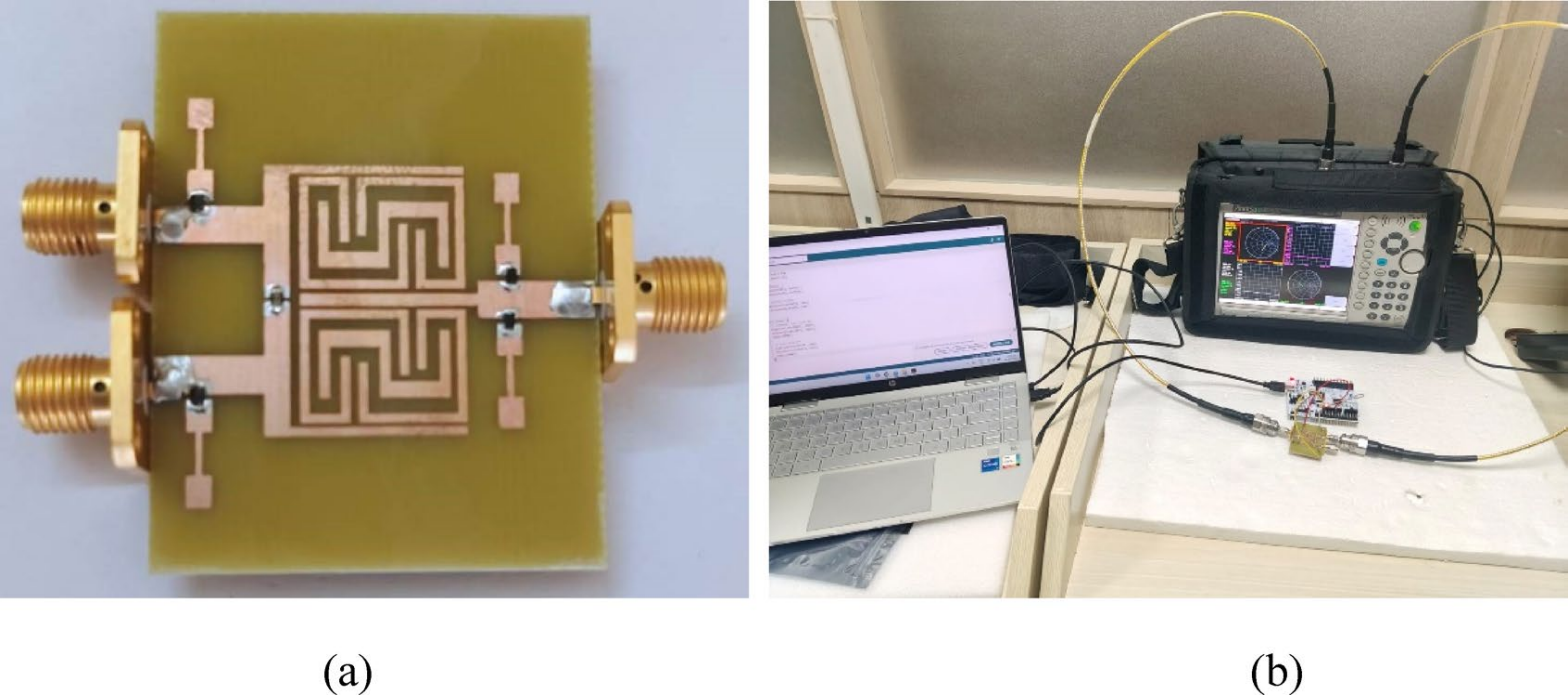
(**a**) fabricated prototype of proposed band tunable filtering power divider. (**b**) Measurement setup for S-parameter with biasing arrangement.

The proposed Band Tunable Filtering Power Divider is fabricated on an FR4 substrate of dielectric constant (εr) = 4.4, loss tangent (tanδ) = 0.02 with 1.6 mm substrate thickness. Figure 9a shows the fabricated prototype of the designed band tunable filtering power divider. Figure 9b shows the biasing and measurement setup. The four RF PIN diodes are biased using an STM32 microcontroller development board. Since the proposed filtering power divider operates in two global switching states (all diodes either ON or OFF), individual bias control is not required. The VCC (5 V) and GND pins of the microcontroller are directly utilized to provide forward and reverse bias conditions, respectively. In the forward-bias state (narrowband mode), a 5 V DC supply is applied, providing approximately 8–10 mA forward current per diode, ensuring low ON-state resistance (≈ 2–5 Ω). In the reverse-bias state (wideband mode), the diodes are effectively at 0 V, behaving as high-impedance elements with low junction capacitance (~ 0.2 pF). This configuration enables stable, repeatable, and real-time electronic switching between wideband and narrowband operating modes without affecting RF performance.

**Fig. 10:**
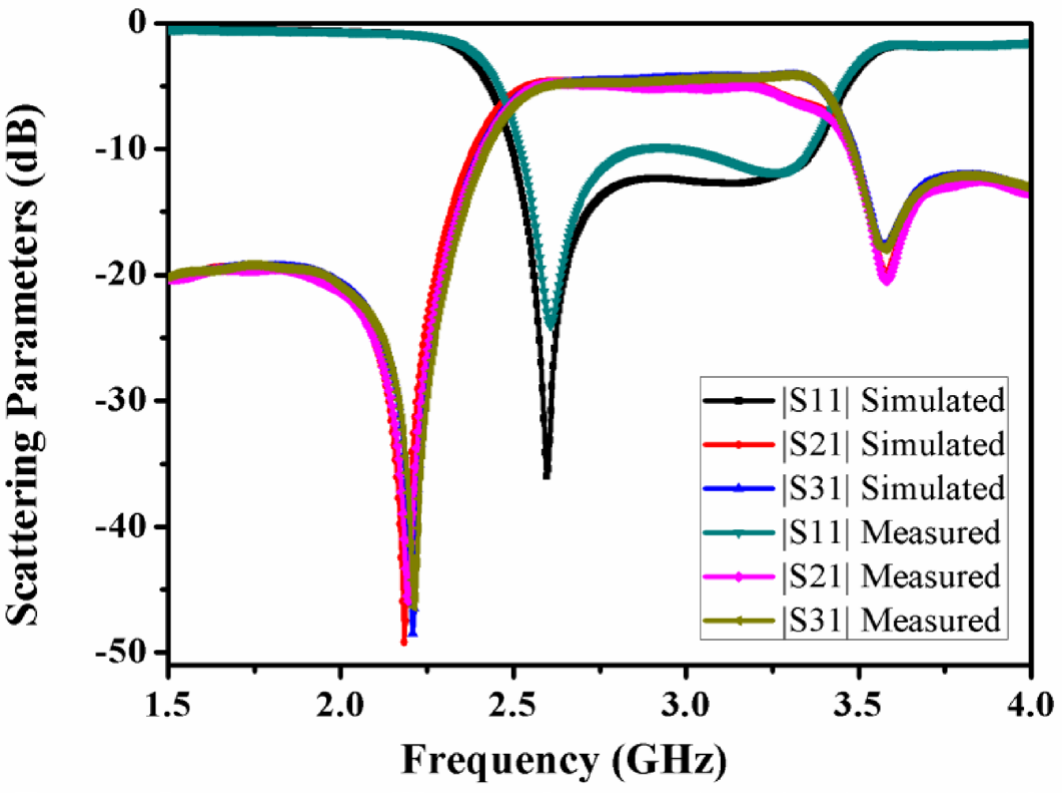
Simulated and measured scattering parameters of FPD when diode OFF.

**Fig. 11:**
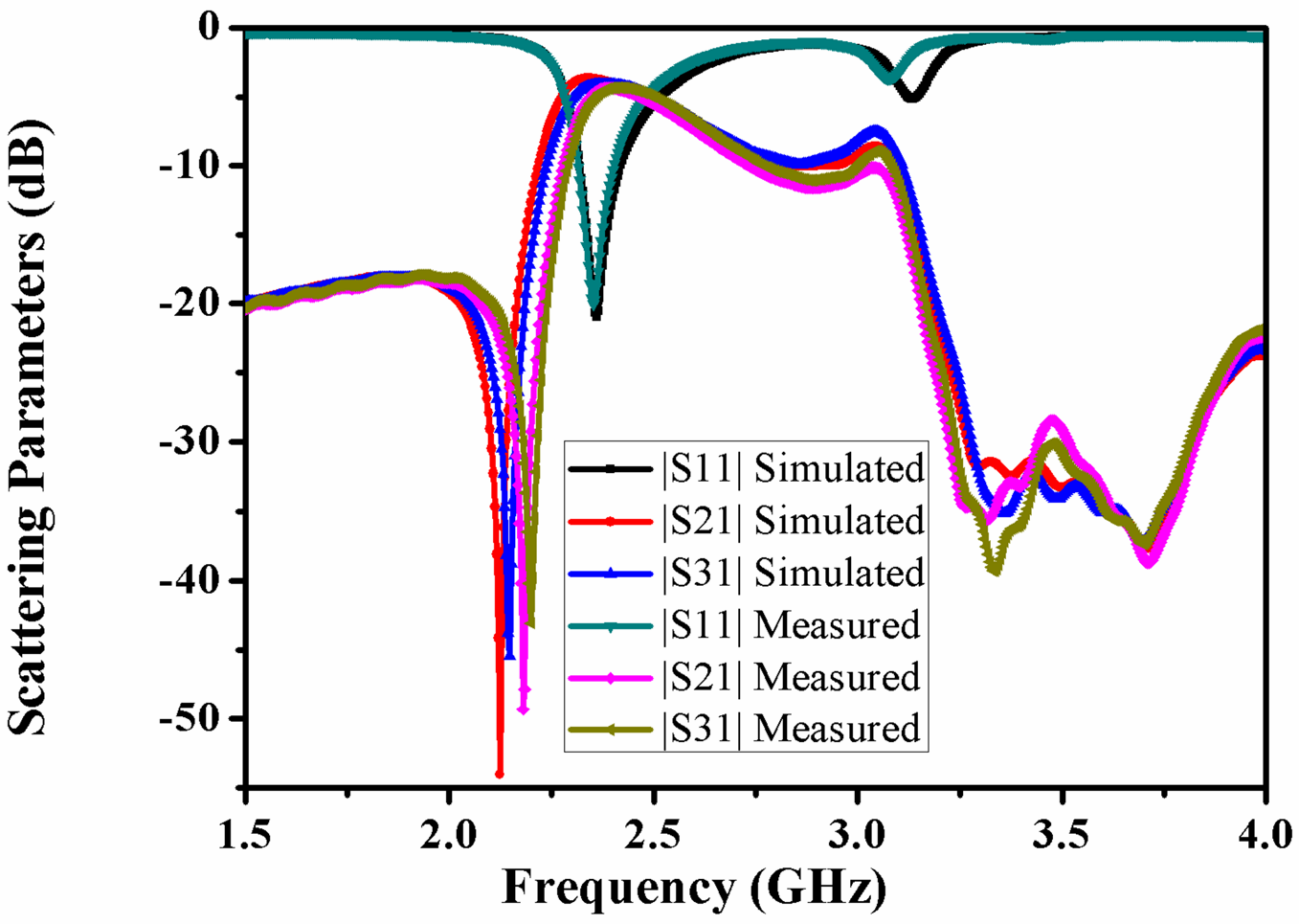
Simulated and measured scattering parameters of FPD when diode ON.

Figure 10. shows input reflection coefficient (S11) and transmission coefficient(S21) of designed filtering power divider in OFF state of four Diodes. When all PIN diodes are in the OFF state, they behave as open circuits, resulting in no changes to the input and output current paths. In this state, the power divider achieves an |S11| value greater than 15.0 dB across the entire operating band and an |S21| value near 4.5 dB, approximating the 3 dB power splitting of a traditional Wilkinson power divider. Furthermore, the device provides isolation levels exceeding 10 dB within the operating band of 2.4 to 3.4 GHz. The center frequency is 2.9 GHz, with a fractional bandwidth of 34.4%. The design is compact, featuring an electrical length of 0.14λ₀ × 0.18λ₀, where λ₀ is the free space wavelength at the resonating frequency of 2.52 GHz. Figure 11. shows about input reflection coefficient (S11) and transmission coefficient(S21) of designed filtering power divider in ON state of four Diodes. When all PIN diodes are in the ON state, the step-impedance resonating stubs connect in parallel to the input and output ports. This configuration allows the rejection band current to flow from the input through the resonating stubs, thereby achieving narrowband filtering power division. In this mode, the power divider exhibits a narrowband resonance at 2.35 GHz. It offers an |S11| value greater than 20.0 dB, indicating excellent return loss performance, and an |S21| value close to 3.5 dB, approaching the ideal 3 dB power splitting of a Wilkinson power divider. The device achieves a 3 dB fractional bandwidth of 4.3%, with isolation exceeding 20 dB within the operating band, ensuring minimal interference and high-quality signal separation.

**Fig. 12:**
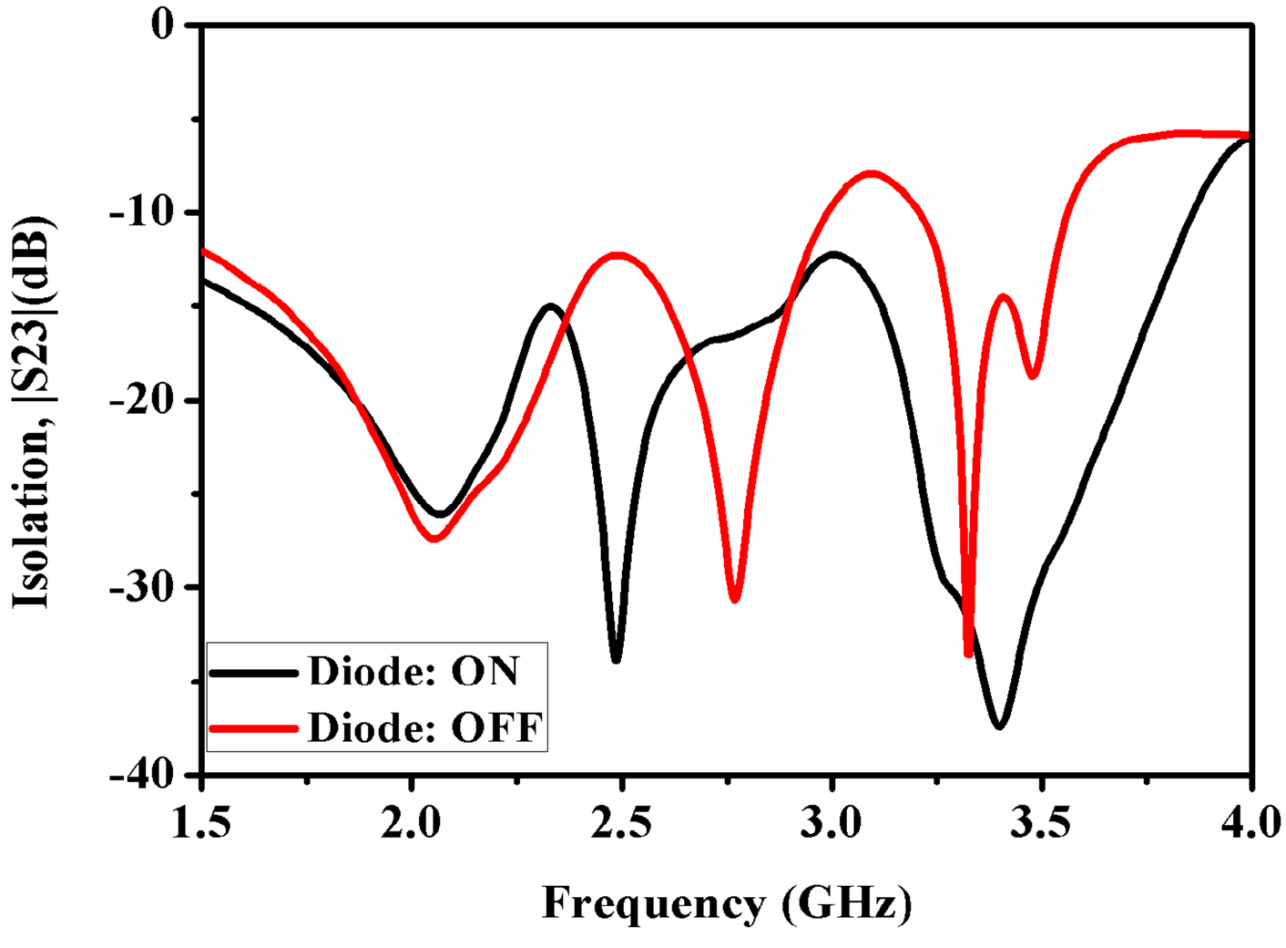
Simulated Isolation of FPD.

Figure 12, explain about isolation in both ON and OFF state of Four PIN diodes. In OFF state the value of isolation is more than 10 dB in the working band 2.4 to 3.4 GHz. Whereas in the ON state, it provides the isolation value more than 15 dB the working band 2.3 to 2.4 GHz.

**Fig. 13:**
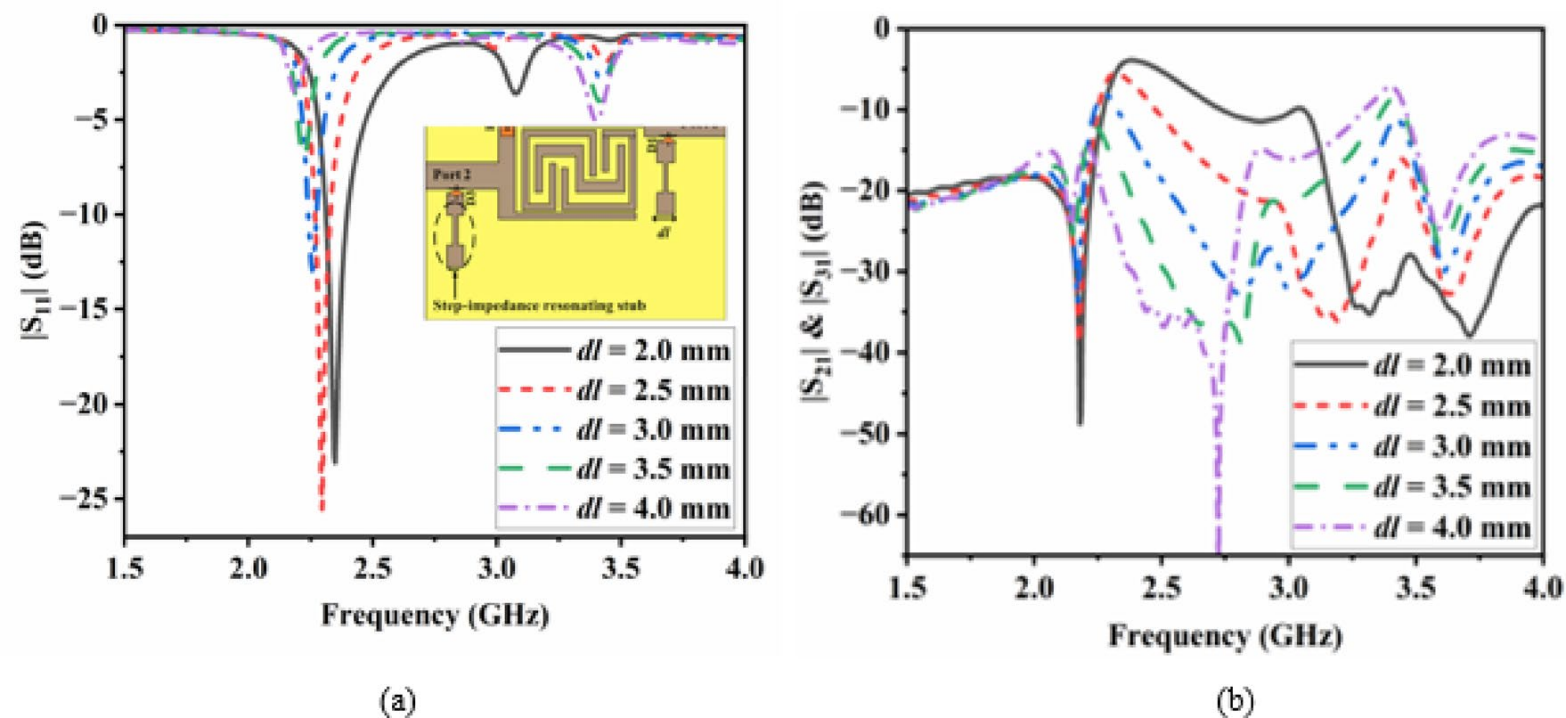
Parametric analysis of step impedance resonating stub, dumbbell width ‘*dl*’ for diode ON state.

Figure 13 shows the variation of reflection coefficient **|S**_**11**_**|**, transmission coefficients **|S₂₁|** and **|S₃₁|** for several values of the design parameter *dl* ranging from 2.0 mm to 4.0 mm. Different colors and line styles (solid, dashed, dotted) are used to differentiate the curves that correspond to each dl value. Figure 13a depicts the resonance frequency shifts towards lower operating frequencies when *dl* is increased because it effectively lengthens the electrical path. Figure 13b depicts about change in transmission coefficients with respect to dumbbell width *dl.* Table 2 presents a comparison between the proposed tunable wide and narrow band FPD with other similar narrow/wideband FPDs that are accessible in the literature and reported in the introduction. The table shows the significant performance characteristics such as electrical size, in-band isolation, centre frequency, tunability, used topology, and fractional bandwidth.

**Table 2:** Performance Summary of the proposed tunable filtering power divider.

Ref. ,year	Topology	Tunability	Fractional bandwith	Center frequency (GHz)	Insertion loss (dB)	Isolation (dB)	Size (λ_o_ × λ_o_)
^8^, 2020	DCRLH	No	24.5%	2.5	3.5	> 15 dB	0.15 × 0.27
^10^, 2019	Coupling-matrix	Yes	10.4% / 15.6%	0.96/1.08	1.4	> 20 dB	**–**
^11^, 2022	All-port reflectionless PD	No	5%	2	2.1	43 dB	0.9 × 3.79
^12^, 2020	Asymmetric coupled line	Yes	57%	1.2	3.17	15 dB	0.165 × 0.067
^25^, 2024	Arbitrary power division	Yes	17.58%	1.6	4.97	20 dB	–
^26^, 2022	Insertion loss control	Yes	3.6%	2.5	1.76	20 dB	0.23 × 0.28
^27^, 2024	Waveguide	Yes	4.5%	3.72	0.24	21 dB	0.72 × 0.62
^28^, 2025	Plasmonic power divider	Yes	29%	5.25	2.0	**–**	1.73 × 1.82
This work	Coupling	Yes	4.3%/ 34.4%	2.35/2.9	1.4/1.6	> 10 dB	0.14 × 0.18

## Conclusion

A compact electronically switchable filtering power divider has been developed using folded resonant structures and diode-controlled step-impedance stubs to achieve dynamic band reconfiguration within a single chip topology. By utilizing reconfigurable capacitive–inductive coupling paths, the design enables selective activation of wideband and narrowband resonant modes without external filter banks or additional switching circuitry. The design proves equal power division and controlled Isolation within a reduced electrical footprint. The measured results confirm the effectiveness of the proposed mechanism: in the wideband (diode-OFF) state, the divider provides a 34.4% fractional bandwidth centered at 2.9 GHz with |S11| > 15 dB, isolation > 10 dB, and insertion characteristics consistent with 3 dB power splitting. In the narrowband (diode-ON) state, the circuit exhibits a resonance at 2.35 GHz with |S11| > 20 dB, isolation > 15 dB, and well-defined filtering selectivity within a 4.3% fractional bandwidth. The overall size of 0.14λ₀ × 0.18λ₀ demonstrates significant miniaturization.

## Data Availability

No datasets were generated or analysed during the current study.
